# Impact of short-term housing temperature alteration on metabolic parameters and adipose tissue in female mice

**DOI:** 10.3389/fendo.2025.1617262

**Published:** 2025-06-24

**Authors:** Henry A. Paz, C. G. Shashank, Lasya Buddha, Tian Lam, Taylor Zhang, Ying Zhong, James D. Sikes, Craig Porter, Reid D. Landes, Roy Morello, Umesh D. Wankhade

**Affiliations:** ^1^ Arkansas Children’s Nutrition Center, University of Arkansas for Medical Sciences, Little Rock, AR, United States; ^2^ Department of Pediatrics, University of Arkansas for Medical Sciences, Little Rock, AR, United States; ^3^ Department of Biostatistics, University of Arkansas for Medical Sciences, Little Rock, AR, United States; ^4^ Department of Physiology & Cell Biology, University of Arkansas for Medical Sciences, Little Rock, AR, United States

**Keywords:** brown fat, ambient temperature, adipose tissue remodeling, maternal metabolism, thermal neutral zone

## Abstract

**Introduction:**

Ambient temperature significantly influences physiological and metabolic processes in rodents, affecting obesity and related disorders. Mice housed below thermoneutral temperatures exhibit increased energy expenditure and sympathetic-driven brown fat activation, whereas thermoneutral housing (~30°C) reduces these responses. This study aimed to determine whether short-term exposure to altered housing temperatures before and during pregnancy induces lasting changes in maternal adipose tissue. We hypothesized that even brief exposure during this critical window could cause persistent structural and molecular alterations in adipose tissue.

**Methods:**

Female C57BL/6J mice were housed at cold (CE, 8°C), thermoneutral (TN, 30°C), or standard room temperature (RT, 22°C) conditions for one week before and throughout pregnancy. All mice were returned to RT post-delivery. Phenotypic assessments—including glucose tolerance, energy expenditure, histology, and proteomics—were performed after lactation.

**Results:**

Temperature exposure did not significantly affect litter size or pup survival. CE-exposed mice showed increased total body weight driven by lean mass gains and reduced fat mass. Adipose tissue showed smaller adipocytes in iWAT and increased vascularity in BAT, though no persistent changes in thermogenic gene expression or glucose homeostasis were observed. Proteomic analysis of iWAT identified 38 differentially expressed proteins, with enrichment of pathways related to mitochondrial function and mTOR signaling.

**Discussion:**

Short-term cold exposure induced lasting histological and proteomic changes in iWAT and BAT without sustained effects on energy metabolism, likely due to reversion to RT and limited sample size.

**Conclusion:**

Brief temperature manipulation around pregnancy can durably alter maternal adipose tissue architecture and molecular signatures, underscoring ambient temperature as an important modulator of maternal metabolic adaptation.

## Introduction

Ambient temperature has a significant impact on numerous physiological and metabolic processes, which can influence the development of obesity and related disorders. Higher ambient temperatures can increase blood flow in the peripheral circulation, leading to excess sweating and heat loss to the environment (1). Conversely, cold ambient temperatures cause peripheral vasoconstriction and activates adaptive thermogenesis, primarily via brown adipose tissue (BAT) (2). Adaptive thermogenesis has garnered renewed interest in this context following the discovery of metabolically active BAT in adult humans (3). The amount of activated BAT was found to negatively correlate with the body mass index (BMI) and body fat percentage of the subjects (4, 5), suggesting a potential role for BAT in human energy metabolism.

Mice are frequently used as model organisms to study the physiological response to environmental stressors such as temperature changes. Compared to thermoneutrally housed mice (~ 30°C in mice, TN), commonly used room temperature of 22°C (RT) can stimulate the activation of BAT and transformation of white adipose tissue (WAT) into brown-like cells (beige) in mice, resulting in increased energy expenditure to maintain body temperature (6, 7). Among other metabolic events, colder ambient temperature can activate the sympathetic nervous system, increasing glucose uptake and lipolysis (8). Conversely, thermoneutrality is the range of ambient temperatures at which an endothermic animal’s body doesn’t need to generate additional heat to maintain core body temperature. At thermoneutrality, the body can maintain its core temperature solely through regulating dry heat loss, such as via skin blood flow (9). Thermoneutral housing in mice leads to a decrease in energy expenditure, glucose uptake, lipolysis, etc. compared to housing at colder temperatures (6, 10). These changes reflect the reduced metabolic demands required for thermoregulation at thermoneutral temperatures. Pregnancy is a critical period of metabolic adaptation, and stress triggers such as diet, exercise can influence maternal energy balance and adipose tissue remodeling (11). We focused on the pre-conception and gestational windows for housing temperature manipulation, as they coincide with oocyte maturation and early fetal development, key stages when environmental cues may induce lasting physiological changes.

In this study, we aimed to establish a foundational model to examine how short-term exposure to different housing temperatures prior to and during pregnancy may influence maternal metabolism, with the broader goal of understanding how the parental thermal environment contributes to offspring metabolic programming. As part of a pilot effort informing future transgenerational studies, we limited temperature exposure to one week prior to conception and the three-week gestational period. We focused on female mice because they remained in the study through gestation and lactation, providing a longer and continuous exposure to the housing temperature compared to males, who were euthanized immediately after breeding. To support successful mating, pregnancy, and lactation—physiological processes sensitive to ambient temperature—all animals were returned to RT following gestation. Accordingly, all phenotypic and molecular assessments were conducted after lactation, under RT conditions. While this design may have attenuated acute temperature effects, it enabled us to examine whether brief periconceptional exposures result in persistent alterations in maternal adipose tissue. These findings serve as an essential foundation for future investigations into how parental temperature exposure shapes offspring metabolic outcomes.

## Materials and methods

### Experimental design

C57BL/6J female mice (5 weeks old) were purchased from the Jackson Laboratory (Bar Harbor, ME, USA). Upon arrival, they were housed at the Arkansas Children’s Research Institute in an AAALAC-accredited facility under controlled humidity and a 12-hour light/dark cycle (lights on from 6:00 a.m. to 6:00 p.m.) at standard room temperature (RT; 22°C). Mice had ad libitum access to a standard chow diet (TD.95092, 18.8% protein, 17.2% kcal fat, 63.9% kcal carbohydrate, 3.8 kcal/gram; Envigo Teklad Diets, Madison, WI, USA) and drinking water. At the end of the study, Mice were euthanized in a rising concentration of CO_2_, followed by tissue collection. This method is consistent with AVMA guidelines for the humane euthanasia of rodents. All animal studies followed local, state, and US Federal regulations and were approved by the UAMS Institutional Animal Care and Use Committee (IACUC) under protocol number AUP# IPROTO202400000007. Veterinarians’ review and approval of the protocol assured that all steps to minimize potential animals’ pain and suffering were taken. The methods used were consistent with the policies of Frontiers in Endocrinology regarding animal experiments.

Due to logistical constraints, the experiment was conducted in three temporally separated cohorts: 15 females in the first, 30 in the second, and 10 in the third, totaling 55 mice. Each cohort was separated by no more than 9 months. After a 2-week acclimatization period at RT, mice were randomized to one of three ambient temperature conditions: RT (22°C, n = 15), cold exposure (CE; 8°C, n = 20), or thermoneutrality (TN; 30°C, n = 20). Individual mice served as the experimental unit. Temperature exposure occurred for a total of 4 weeks: 1 week prior to mating and 3 weeks during gestation. Mice were singly housed in temperature-controlled cabinets (Cat# RIS52SD, Powers Scientific, Pipersville, PA) during these periods. The cages were rotated daily within the central columns of the cabinets to avoid positional bias. After the initial 1-week exposure to their assigned temperature, females were temporarily returned to RT for mating with males, which had been preconditioned for 1 week at the same temperature as their assigned female partner. Male mice were not included in this analysis because they were euthanized at 12 weeks of age, immediately following breeding, and had only been exposed to housing temperature manipulation for one week prior to mating. This limited exposure period made direct comparison to female mice, who experienced a longer and continuous exposure through gestation, inappropriate for meaningful analysis. Pregnancies were not strictly timed, but monitored based on the presence of vaginal plugs and weight gain. Following confirmation of mating, females were returned to their respective temperature-controlled cabinets for the 3-week gestational period.

**Figure 1:**
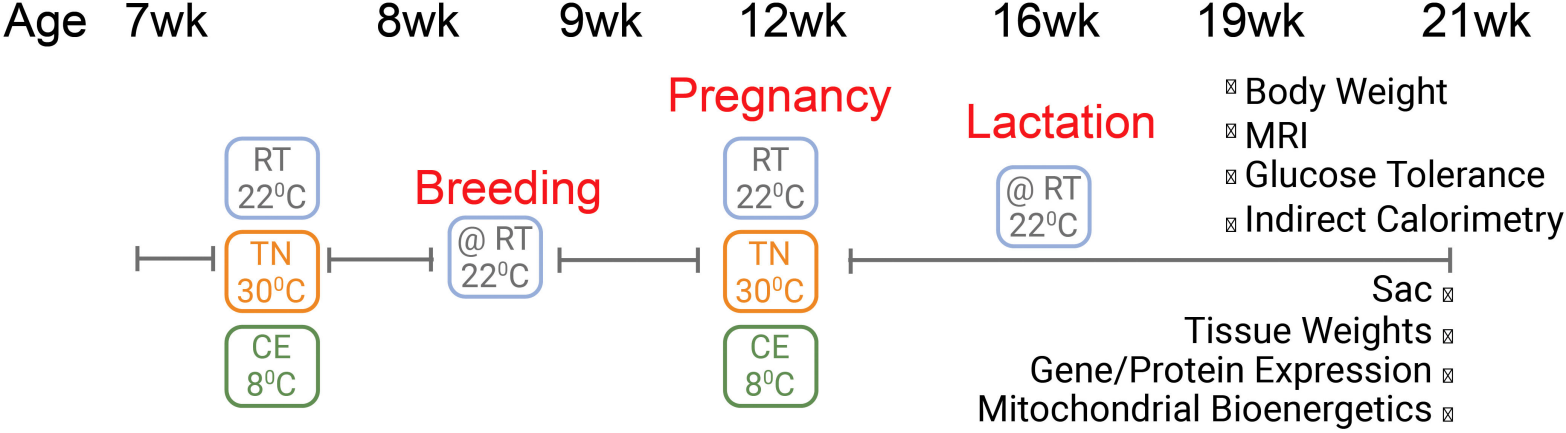
Schematic representation of experimental design. Female mice were exposed to cold (8°C, CE), room (22°C, RT), or thermoneutral (30°C, TN) temperature for one week prior to conception and for three weeks during gestation. After four weeks of lactation, mice underwent several metabolic assays and were eventually euthanized at 21 weeks of age. All the assays were performed when mice were housed at RT.

To ensure optimal conditions for pregnancy, delivery, and lactation, processes sensitive to ambient temperature, all females were returned to RT immediately after gestation and remained there through pup delivery, lactation, and weaning. After weaning, mothers continued to be housed at RT for an additional 5 weeks (21 weeks of age) prior to euthanasia and tissue collection (**Figure 1**). Most of the assays/tests reported are done from age of week 17 to week 21. This design reflects a conservative and feasibility-focused approach, prioritizing animal welfare and reproductive success while still enabling us to assess the long-term impact of early temperature exposure.

While housing temperature manipulations occurred one week prior to conception and continued throughout the gestational period, all phenotypic assessments—including metabolic testing, histology, gene expression, and proteomic analyses—were conducted only after the completion of lactation. No data were collected during pregnancy or lactation. This design allowed us to investigate whether brief thermal exposures around the periconceptional window could elicit long-term alterations in maternal adipose tissue, independent of direct gestational or lactational influence.

### Glucose tolerance test

For the GTT, a subset of 18-week-old female mice (n = 6 per group) were fasted for 14 hours. The next morning, they were injected intraperitoneally with glucose (2 mg/kg total body weight). Tail blood was collected prior to glucose injection (0 min) and at 15, 30, 60, and 90 minutes for glucose measurement using a Bayer Contour Next Meter and glucose strips (Cat# 32006-06).

### Body composition

Body composition was measured at 19 weeks of age using the EchoMRI NMR analyzer (EchoMRI, Houston, TX), which provided non-invasive estimates of lean and fat mass.

### Body temperature

Body temperature was recorded weekly using implantable BMDS IPTT-300 transponders (Avidity Science, Waterford, WI), which also served as individual animal identifiers. To minimize stress during gestation, body temperature measurements were paused during pregnancy.

### Indirect calorimetry

Female mice at 17-week of age underwent energy expenditure assay. Mice were individually housed for 5 consecutive days in specialized cages that allowed VO_2,_ VH_2_O and VCO_2_ to be continuously measured to calculate energy expenditure (Sable Systems International, Las Vegas, NV, USA). During this time, food and water intake, and activity were continuously recorded. During metabolic and behavioral phenotyping, animals were housed in environmental cabinets to control ambient temperature at 22°C for all the groups. Data were analyzed using Sable Systems International ExpeData software (Version 1.9.27). Data from three consecutive 12:12 hour light-dark cycles were averaged to provide daily values. Hourly rates of energy expenditure were calculated from VO_2_ and VCO_2_ using the Weir equation:


$$EE\;\left(\frac{kcal}{hr}\right)\;=60\times \left(0.003941\;\times \;VO_{2})+(0.001106\;\times \;VCO_{2}\right)$$

Respiratory exchange ratio (RER) was calculated using the ratio of carbon dioxide produced (VCO_2_) to oxygen consumed (VO_2_), calculated as RER = VCO_2_/VO_2_. This value reflects substrate utilization, with RER values approaching 1.0 indicating predominant carbohydrate oxidation and values near 0.7 indicating predominant fat oxidation. Total daily energy expenditure (TEE) was calculated as the sum of the average rate of energy expenditure (EE; kcal/hour) for both the light and dark cycle times 12, summed and averaged across the final 4 days of the experimental period.

### Oroboros

Interscapular BAT and inguinal WAT (iWAT) depots were excised and weighed upon euthanization with scissor and forcep. Tissue was then immediately placed in ice-cold BIOPS (Biopsy preservation solution) preservation buffer (10 mM Ca-EGTA buffer, 0.1 µM free calcium, 20 mM imidazole, 20 mM taurine, 50 mM K-MES, 0.5 mM DTT, 6.56 mM MgCl_2_, 5.77 mM ATP, 15 mM phosphocreatine, pH 7.1) for respirometry measures. NADH-linked respiration in the leak (uncopled state) was assayed in the presence of pyruvate (5mM) and malate (2mM), and then following the subsequent addition of glycerol-3-phosphate (10mM). The UCP1 inhibitor GDP (20mM) was then added to alow quantification of UCP1-dependent leak respiration. The coupling control ratio for GDP was also calculated to provide a qualitive index of mitochondrial coupling control.

### Histology

Adipose tissue depots were fixed in 10% formalin overnight, dehydrated in 70% ethanol, and embedded in paraffin. Six-micrometer-thick section from iWAT and BAT were stained with hematoxylin and eosin (H&E). Stained slides were viewed and images were captured with Nikon ECLIPSE Ti2 at 20X magnification (Nikon Instruments Inc. Melville, NY, U.S.A). An unbiased selection of images was performed by a third-party individual, blinded to the experimental conditions.

### Real-time RT-PCR

Total RNA was isolated from adipose tissue using RNeasy mini columns (QIAGEN, Valencia, CA) including on-column DNase digestion. Five hundred nanograms of total RNA was reverse transcribed using the iScript cDNA synthesis kit (BioRad, Hercules, CA). Realtime PCR analysis was performed as described previously using an ABI Prism 7500 Fast instrument (Carlsbad, CA). Gene specific primers were designed using Primer Express Software (**Supplementary Table 1**) Relative mRNA levels were quantified using the 2^−ΔΔCT method, with 18S rRNA serving as the housekeeping gene. The 22°C (RT) group was used as the reference control for all relative comparisons.

### Quantitative proteomics

Liquid N_2_ snap-frozen iWAT tissue was used for proteomics. Total protein from tissue was reduced, alkylated, and purified by chloroform/methanol extraction prior to digestion with sequencing grade modified porcine trypsin (Promega, Madison, WI, USA). Tryptic peptides were then separated by reverse phase XSelect CSH C18 2.5 um resin (Waters, Milford, MA) on an in-line 150 x 0.075 mm column using an UltiMate 3000 RSLCnano system (Thermo). Peptides were eluted using a 60 min gradient from 98:2 to 65:35 buffer A:B ratio (Buffer A = 0.1% formic acid, 0.5% acetonitrile; Buffer B = 0.1% formic acid, 99.9% acetonitrile). Eluted peptides were ionized by electrospray (2.4kV) followed by mass spectrometric analysis on an Orbitrap Exploris 480 mass spectrometer (Thermo). To assemble a chromatogram library, six gas-phase fractions were acquired on the Orbitrap Exploris with 4 m/z DIA spectra (4 m/z precursor isolation windows at 30,000 resolution, normalized AGC target 100%, maximum inject time 66 ms) using a staggered window pattern from narrow mass ranges using optimized window placements. Precursor spectra were acquired after each DIA duty cycle, spanning the m/z range of the gas-phase fraction (i.e. 496–602 m/z, 60,000 resolution, normalized AGC target 100%, maximum injection time 50 ms). For wide-window acquisitions, the Orbitrap Exploris was configured to acquire a precursor scan (385–1015 m/z, 60,000 resolution, normalized AGC target 100%, maximum injection time 50 ms) followed by 50x 12 m/z DIA spectra (12 m/z precursor isolation windows at 15,000 resolution, normalized AGC target 100%, maximum injection time 33 ms) using a staggered window pattern with optimized window placements. Precursor spectra were acquired after each DIA duty cycle.

### Proteomics data analysis

Following acquisition, data were searched for proteins using an empirically corrected library and a quantitative analysis was performed to obtain a comprehensive proteomic profile. Proteins were identified and quantified using EncyclopeDIA and visualized with Scaffold DIA using 1% false discovery thresholds at both the protein and peptide level (12). The UniProtKB Mus musculus database was used for the database search. Protein exclusive intensity values were assessed for quality and normalized using ProteiNorm (13). The data was normalized using Cyclic Loess and statistical analysis was performed using Linear Models for Microarray Data (limma) with empirical Bayes (eBayes) smoothing to the standard errors (14). Proteins with an FDR adjusted *p*-value <0.05 and a log fold change >1.5 were considered to be significant. Gene ontology enrichment analysis was performed to determine significantly enriched biological processes using the online tool GOrilla (15). The background gene list used for analysis included only those proteins detected in the proteomic experiment, and a statistical threshold of 1 × 10–^3^ was used to determine significantly enriched processes (16).

### Statistical analysis

Data are presented as a box plot, in which the box represents the interquartile range. A horizontal line goes through the box at the median. Multiple comparisons between experimental conditions were adjusted for multiple tests, using Dunnett’s or Sidak’s where appropriate. ANCOVA analyses were performed in R Studio (Version 1.4.1717, RStudio, PBC, Boston, MA). Statistical significance was set at *P* < 0.05. Though no inclusion/exclusion criteria for data points were set *a priori*, all mice provided data, and all data were used in the analyses.

The animals used in this experiment were part of a larger experiment that involved their litters. As such, original sample size considerations were based on the larger experiment. However, we computed the difference between a pair of temperature groups (e.g., CE vs TN) detectable with approximately 0.80 power with a 0.05 significance level *t*-test conducted within the ANOVA for three sample sizes: with 18 same-sex mice per group the detectable effect size was 0.95 SD; with 9 per group, 1.40 SD; and with 6 per group, 1.75 SD. Here, SD is the within-group standard deviation, assumed constant among the 3 groups. A primary outcome measure for this portion of the larger study is total body weight.

## Results

### Pre-conception and gestational housing temperature and its impact on litter size and survival

**Figure 2:**
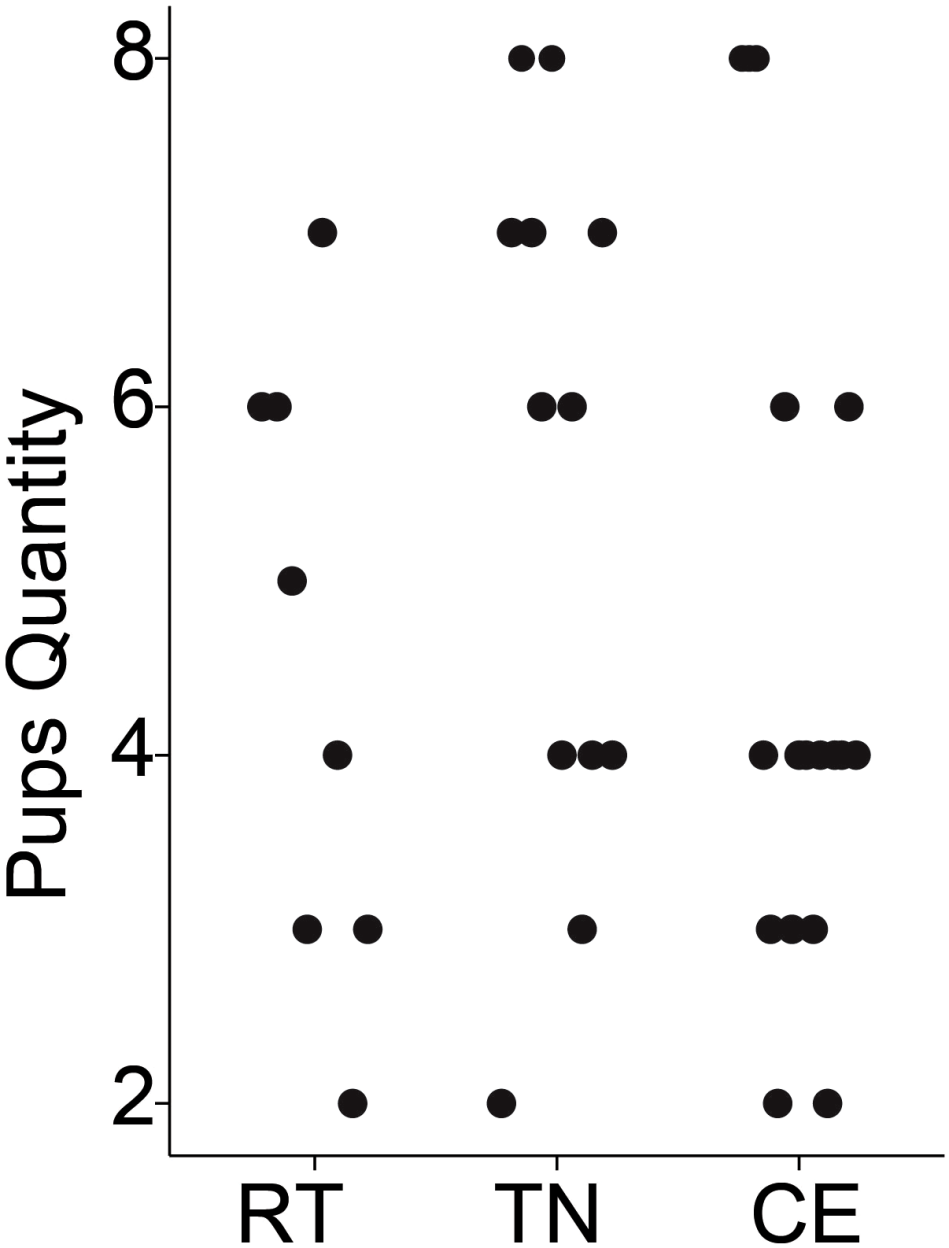
Litter sizes did not differ by housing temperature. Litter sizes are plotted by treatment: room temperature (RT [20°C], n = 10 breeding pairs), thermoneutral (TN [30°C], n = 15 breeding pairs), and cold exposure (CE [8°C], n = 15 breeding pairs). No significant differences in breeding performance were found among the temperature groups.

The impact of pre-conception (1 week) and gestational housing temperature on litter size and pup survivability was evaluated. No pups from any of the litters died before weaning. There was no evidence to suggest that average litter size differed significantly among dams exposed to RT, TN, or CE for 1 week prior to conception and 3 weeks during gestation. The mean litter sizes were 2.4 for RT, 3.0 for TN, and 2.6 for CE, with a pooled standard deviation of 1.38 (**Figure 2**). To assess whether variation in litter size could have influenced maternal outcomes, we conducted an exploratory correlation analysis between individual litter sizes and key metabolic measures (including total body weight, fat mass, food intake, and energy expenditure). No significant associations were observed, suggesting that differences in litter size did not systematically affect the primary results.

### Metabolic phenotype in mice following short-term housing temperature manipulation

**Figure 3:**
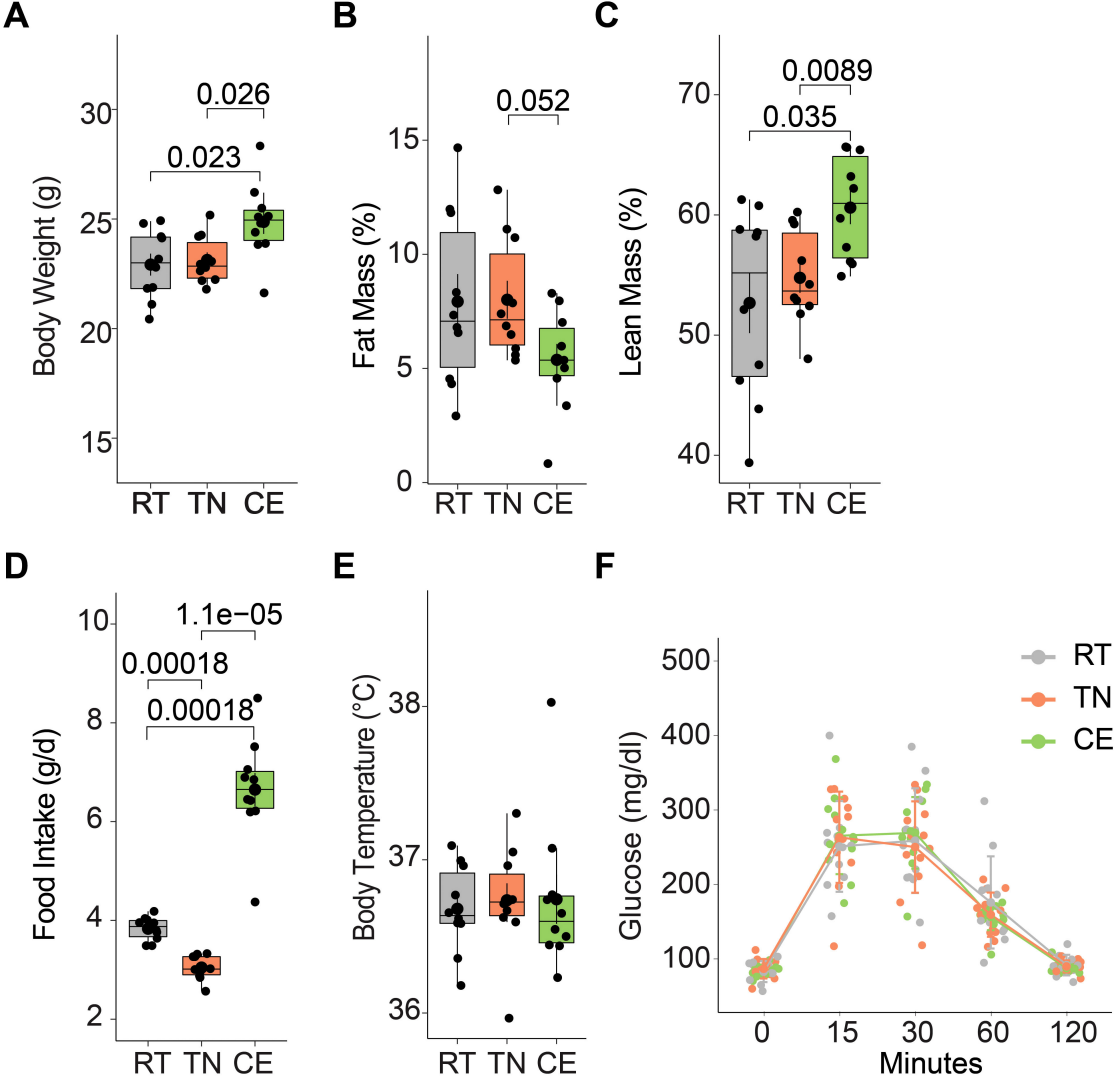
Short-term exposure to cold and thermoneutral temperature altered the total body weight, body composition, and food intake. Female mice were exposed to room (22°C, RT), thermoneutral (30°C, TN), or cold (8°C, CE) temperature for a total of four weeks (one week prior to conception and three weeks during gestation). The CE group showed increased total body weight **(A)** with lower fat mass **(B)** and higher lean mass **(C)** when compared to the other groups. Change in total body weight was accompanied by increased food intake **(D)**. Daily body temperature **(E)** and glucose tolerance **(F)** did not differ among groups. Sample sizes were n = 10 mice/group, except for glucose tolerance, where n = 6 mice/group. Data are presented as a box plots, with the box representing the interquartile range and a horizontal line indicating the median.

The impact of environmental temperature fluctuations on physiological processes is critical in understanding metabolic adaptations. The effects of short-term cold and thermoneutral temperature exposure on total body weight, body composition, and food intake were assessed in female mice. Over a four-week period (including one week prior to conception and three weeks during gestation), the CE group exhibited a significant increase in total body weight compared to the TN and RT groups (**Figure 3A**). This weight gain was accompanied by a decrease in fat mass and an increase in lean mass (**Figures 3B,C**). Additionally, food intake was elevated in the CE group compared to the RT and TN groups (**Figure 3D**). Daily body temperature and glucose tolerance (at 17^th^ week) did not show significant differences between the groups (**Figures 3E, F**).

### Energy expenditure in female mice following short-term manipulation of housing Temperature

**Figure 4:**
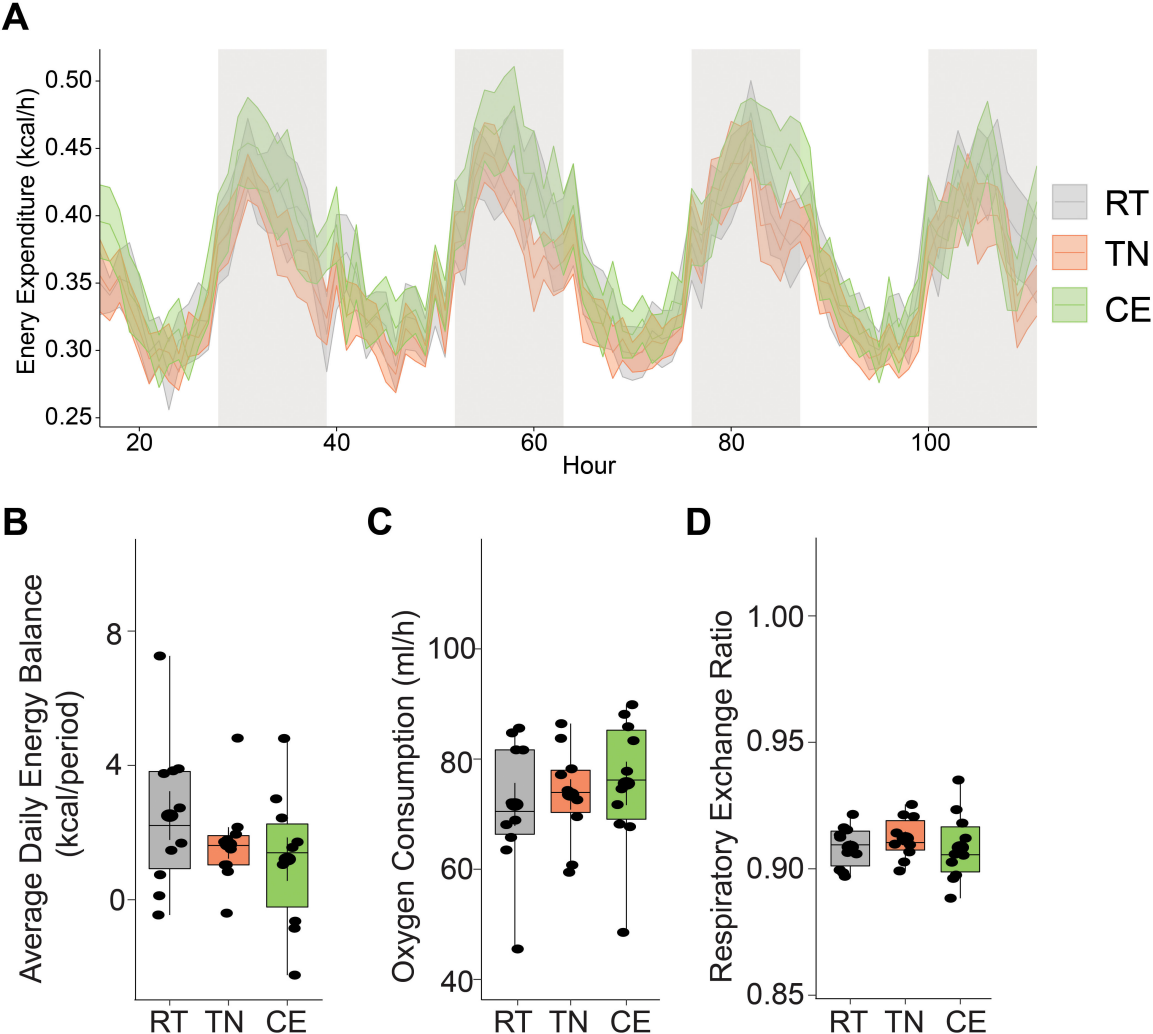
Short-term exposure to cold and thermoneutral temperature did not significantly alter energy expenditure in female mice. Indirect calorimetry was performed on mice at room temperature condition. Energy expenditure was measure during light and dark phases **(A)**. There were no significant differences in energy balance **(B)**, oxygen consumption **(C)**, and respiratory exchange ratio **(D)** among the groups. RT = room temperature (22°C), TN = thermoneutral (30°C), CE = cold exposure (8°C). Sample sizes were n = 6 mice/group and data are presented as a box plots, with the box representing the interquartile range and a horizontal line indicating the median **(B–D)**.

Thermoneutral and cold housing temperatures are known to impact energy expenditure in rodents (17, 18). We investigated the impact of previous short-term exposure (1 week prior to breeding, and 3 week during gestation) to cold and thermoneutral housing temperatures on energy expenditure and energy balance in female mice while housed at 22°C. Previous randomization to thermoneutral and cold temperature housing intermittently did not significantly alter energy expenditure or balance in mice when compared to RT or between the three (all 3 *P*>0.05). Indirect calorimetry was performed on these mice at room temperature condition (RT n = 6, TN n = 6, CE n = 6) (**Figures 4A–D**).

### Impact of transient changes in housing temperature on adipose tissue

**Figure 5:**
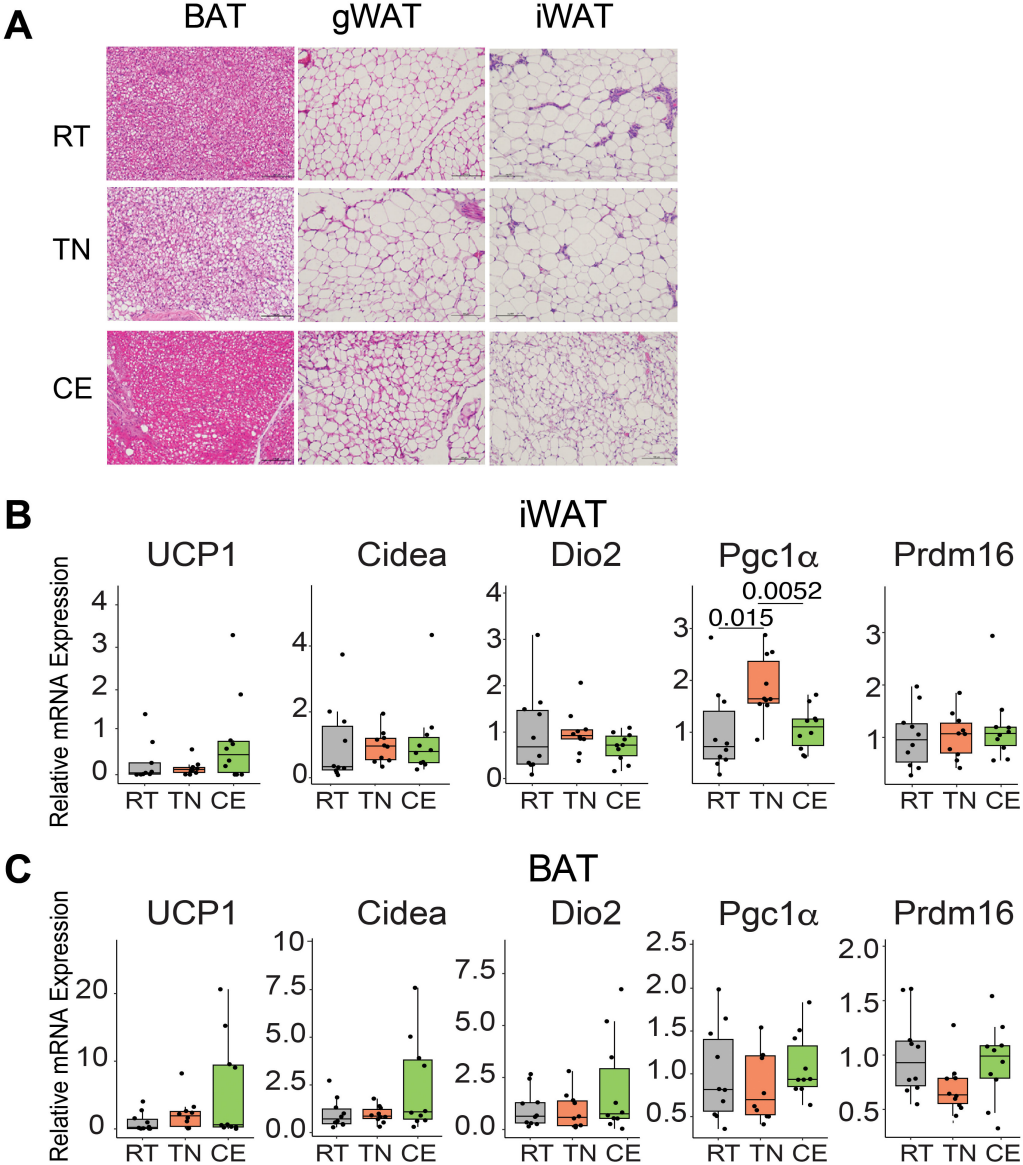
Housing of mice intermittentaly at thermoneutral and cold temperature induced changes in the adipose tissue. Female mice were exposed to room (22°C, RT), thermoneutral (30°C, TN), or cold (8°C, CE) temperature for a total of four weeks (one week prior to conception and three weeks during gestation). CE mice showed smaller sized adipocytes in iWAT and more vascularized BAT, whereas TN mice displayed lipid-laden BAT **(A)**. An unbiased selection of images was performed by a third-party individual, blinded to the experimental conditions, from a cohort of n = 6 mice/group. In iWAT **(B)** and BAT **(C)**, brown specific transcripts were unchanged. 2^−ΔΔCT method and 18S rRNA as the housekeeping gene were used for analysis. 22°C (RT) group served as the reference control for relative comparisonsSample sizes were n = 10 mice/group and data are presented as a box plots, with the box representing the interquartile range and a horizontal line indicating the median **(B, C)**. Statistical significance was declared at p<0.05.

Variation in ambient temperature influences the morphology and physiology of adipose tissue. We examined the effect of housing temperature changes on the adipose tissue morphology and transcript profile in female mice. Short-term cold exposure resulted in the presence of smaller-sized adipocytes in iWAT and enhanced vascularization in BAT, especially as compared to mice housed at thermoneutral temperatures. Conversely, thermoneutral conditions led to lipid-laden BAT (**Figure 5A**).

In female mice, short-term exposure to different temperatures for 4 weeks did not induce significant alterations in the expression of brown fat-specific genes (*Ucp1*, *Cidea*, *Dio2*, *Pgc1a*, and *Prdm16*) in iWAT, with the exception of *Pgc1a* (**Figure 5B**). Specifically, *Pgc1a* exhibited increased expression in the iWAT of female mice housed at TN compared to both RT and CE mice. Interestingly, in BAT of cold-exposed female mice, *Ucp1*, *Dio2*, and *Cidea* showed a trend of higher expression compared to TN and RT-housed mice. (**Figure 5C**). These findings underscore the influence of environmental temperature on adipose tissue structure and gene expression.

### Housing temperature and thermogenic capacity of BAT and iWAT

**Figure 6:**
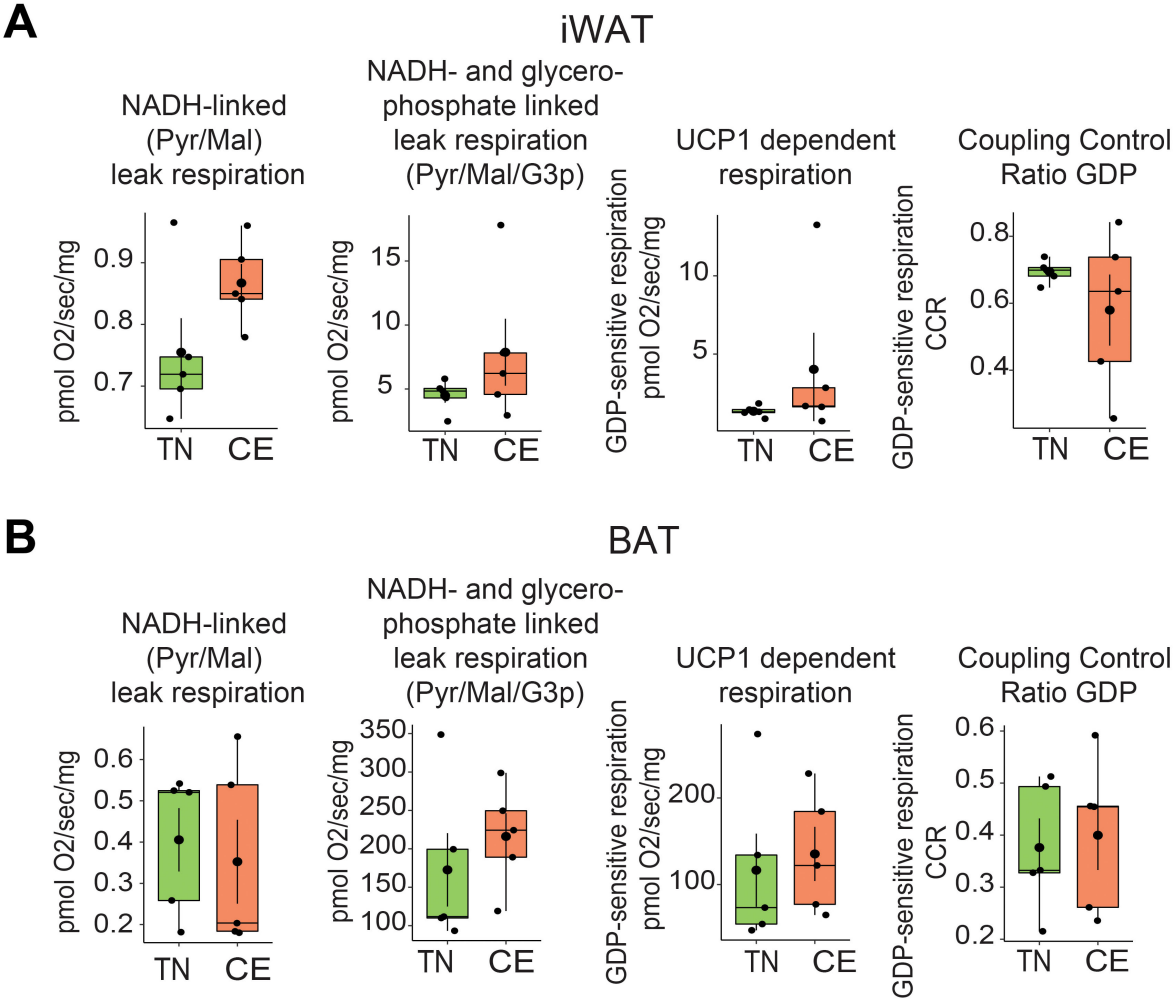
Short-term exposure to cold and thermoneutrality did not alter the thermogenic capacity of BAT and iWAT. NADH-linked (Pyr/Mal) leak respiration, NADH- and glycerophosphate-linked leak respiration (Pyr/Mal/G3p), UCP1-dependent respiration, and associated coupling control ratio for GDP in iWAT **(A)** and BAT **(B)** were measured in mice housed at room temperature conditions at 18 weeks of age in female mice. Sample sizes were n = 5 mice/group and data are presented as a box plots, with the box representing the interquartile range and a horizontal line indicating the median.

The impact of previous exposure to cold on the thermogenic capacity of BAT and iWAT was investigated in permeabilized tissue. Short-term exposure to cold did not significantly alter the respiratory capacity of BAT and iWAT. The NADH-linked (Pyr/Mal) leak respiration, NADH- and glycerophosphate-linked leak respiration (Pyr/Mal/G3p), UCP1-dependent respiration, and associated coupling control ratio for GDP were measured in iWAT and BAT of female mice (**Figures 6A, B**). The measurements were made at 18 weeks of age (after being at RT during the lactation). There were no significant differences in any of the measured parameters between mice that were previously exposed to cold and those that were not. These findings have implications for the study of metabolic disorders and the management of obesity, as the thermogenic capacity of adipose tissue is an important factor in energy expenditure and regulation of total body weight.

### Short-term exposure to cold housing temperature altered the iWAT proteome

**Figure 7:**
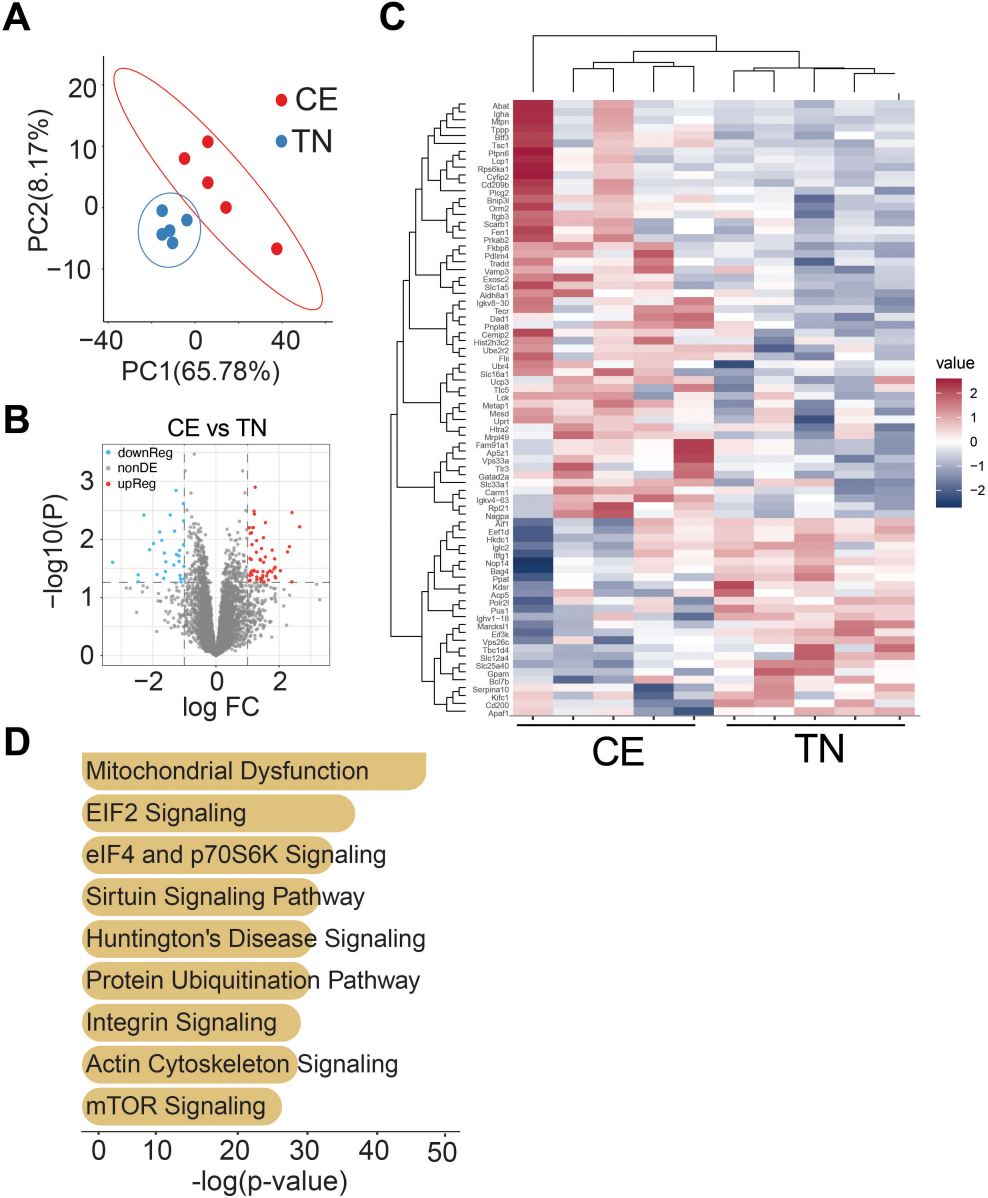
Previous exposure to cold temperature resulted in long-lasting changes in the proteome of iWAT. Principal co-ordinate analysis shows clustering of proteins based on temperature conditions in female mice iWAT **(A)**. Volcano plot showing the effect of temperature on expression of iWAT proteins **(B)**. Out of 410 proteins in iWAT, 38 were found to be differentially regulated (p<0.05, and log_2_FC > 1.5fold). Heatmap reflecting differentially regulated proteins **(C)**. Ingenuity pathway analysis of differentially expressed genes **(D)**. Sample sizes were n = 5–6 mice/group.

The impact of short-term exposure to cold and thermoneutral temperature on the proteome of iWAT was investigated in female mice. The results indicate that previous exposure to cold resulted in long-lasting changes in the proteome of iWAT. Principle co-ordinate analysis showed clustering of samples based on temperature conditions in iWAT. The volcano plot demonstrated the effect of temperature on the expression of iWAT proteins. In iWAT, a total of 38 proteins out of 410 were differentially regulated (**Figures 7A, B**).

The heatmap showed the differentially regulated proteins in iWAT (**Figure 7C**). The results suggest that previous exposure to cold led to long-lasting changes in the proteome of iWAT in female mice. Ingenuity pathway analysis of differentially expressed genes were primarily associated with pathways related to mitochondrial dysfunction, ELF2 and ELF4 signaling, and mTOR (**Figure 7D**). Proteins associated with each pathways are listed in **Supplementary Table 2**. These findings suggest that housing temperature can significantly impact the proteome of adipose tissue, which may have implications for the management of metabolic disorders such as obesity.

## Discussion

Our study examined the effects of short-term CE and TN compared to RT in female mice immediately prior to and during pregnancy, exploring its long-term impact on body composition, food intake, adipose tissue proteome, energy expenditure, and mitochondrial function. Short-term cold exposure (4 weeks) in female mice resulted in greater total body weight due to an increase in lean mass and a concomitant reduction in fat mass. Mice previously intermittently exposed to cold exhibited smaller-sized adipocytes in WAT and more vascularized BAT, coupled with long-lasting changes in the adipose tissue proteome. Interestingly, we found no evidence that previous short-term cold exposure exerts long-lasting control on the thermogenic capacity of adipose tissue in mice; however, only large differences would have been detectable with the sample size in our study. Environmental temperature can have a significant impact on the reproductive performance of rodents (19). Factors such as temperature, humidity, light cycle, and stressors like noise and odors can all affect breeding outcomes. Maintaining temperatures of 18-23°C with 40-60% humidity, along with a 14-hour light/10-hour dark cycle, is recommended to support optimal breeding (20–22). A study by Barnett et al. demonstrated that breeding at lower temperatures, such as -3°C, delayed breeding and led to higher mortality between birth and weaning, compared to breeding at higher temperatures such as 10°C and 21°C (23). On the other hand, another study found that a temperature range of 22°C to 28°C was optimal for high-quality embryos, with sperm progressive motility remaining high within this range (20). The study suggested that mice housed at higher temperatures, such as 28°C, could maintain reproductive fitness at high levels (20). Although we evaluated the effects of short-term housing temperature on maternal metabolic outcomes, assessment of postnatal growth or metabolic parameters in the offspring is under investigation and part of future publications. While offspring sex ratio was analyzed and found to be unaffected by maternal temperature exposure, offspring growth and metabolic health are currently under investigation and will be addressed in a separate manuscript. This distinction reflects the pilot nature of the current study, which was designed to characterize maternal responses as a foundation for future work exploring transgenerational metabolic programming. Our study aligns with previous research, finding no evidence that intermittant exposure to varying temperatures before conception and during gestation affects litter size or pup survivability.

Our investigation into the long-term metabolic consequences of short-term temperature manipulation reveals intriguing nuances. The observed increase in total body weight in cold-exposed mice echoes findings from studies linking colder environments to weight gain (24, 25). This is most likely due to higher food intake in response to increased energy demands, which leads to better utilization of energy substrates as a result of the cold stress. Overall weight gain in CE female mice in the current study is accompanied by a decrease in fat mass and an increase in lean mass, consistent with earlier studies on cold-induced weight gain (24, 25). A study in which rats were moved from 28°C to 20°C showed increased weight gain and subcutaneous fat mass, suggesting a direct effect of a cooler housing temperature in promoting weight gain and inducing further adiposity in obese rats (24). However contrary to findings from the current study, this group did not report increase in food intake in these mice. When mice are exposed to cold, one explantion for weight gain without increased food intake is the elongation of their intestines (24). This elongation increases surface area, enhancing fatty acid absorption and nutrient permeability. Cold exposure also raises intestinal paracellular permeability, possibly due to enterocyte hyperplasia, which results in more tight junctions for nutrient passage (26). These adaptations suggest that mice activate compensatory mechanisms to optimize nutrient absorption and balance energy needs, counteracting the higher energy expenditure induced by cold exposure.

Contrary to our hypothesis, the lack of significant changes in glucose tolerance or daily body temperature in the mice suggests three possible explanations. We did not assess gestational hormone levels, however we measured circulating glucose and insulin at euthanasia and found no significant differences among groups. First, the mice may possess a notable metabolic adaptability that enables them to adjust to temperature fluctuations effectively. Second, the acute and short-term nature of the temperature exposure might not have been intense or prolonged enough to induce measurable shifts in glucose homeostasis. Lastly, the 8–9 week washout period between temperature exposure and metabolic measurements may have diminished any phenotypic impact, potentially masking subtle effects. Previously, it is demonstrated that short-term cold exposure improves glucose homeostasis despite exacerbating diet‐induced obesity in mice housed at thermoneutrality aided by browning of WAT (27). The absence of sustained changes in energy expenditure or balance in our study may be due to two main factors. First, the brief duration of exposure to varying ambient temperatures might have been insufficient to produce lasting effects. Second, since the metabolic assays were performed after the mice had spent considerable time at room temperature, the prolonged exposure to RT could have mitigated or nullified the effects that the prior cold and thermoneutral exposures had on the mice. Sadler et al. reported that transitioning from 24°C to 30°C reduced total energy expenditure in both male (25%) and female (16%) mice, which was attributable to lower basal energy expenditure in males (36%) and females (40%) (18). At thermoneutrality, food intake is reduced because of less energy demands, and vice-versa at colder temperature, there is a higher food intake accompanied by higher energy expenditure (28, 29). As mentioned earlier, the increase in food intake in mice was accompanied by increased weight gain but did not lead to any alterations in energy expenditure or balance. The lack of discernible differences in many of the parameters in our study can be attributed to the smaller sample size, which was not powered to detect small differences.

Additional exploration of adipose tissue provided further insight into the adaptive responses to housing temperature changes. The smaller adipocyte size in cold-exposed mice aligns with previous studies indicating enhanced lipolysis and thermogenic activity in response to cold, leading to a reduction in adipocyte size. However, a study reported that short-term cold exposure actually increased adiposity despite enhancing thermogenic activity, and the adipocytes of inguinal fat were significantly larger in short term CE-treated mice, while epididymal adipocytes were smaller, suggesting increased adipogenesis in the latter and indicating a tissue-specific response to cold exposure (30). Similarly, the increasing trend in *Ucp1* expression in iWAT and BAT of cold-exposed mice is consistent with the classical response to cold exposure, where increased *Ucp1* expression facilitates heat production through uncoupling of mitochondrial respiration. Previously it was demonstrated that thermoneutrality decreases the thermogenic program (genes such as *Ucp*1, *Cidea*, *Prdm16*, *Pgc1a*, etc) and promotes adiposity in high-fat diet-fed mice (28). When mice were switched to room temperature (22°C) from thermoneutral temperature (28°C), *Ucp1* expression in WAT increased, in comparison to the mice that stayed at thermoneutrality (31). These findings highlight the temperature-dependent regulation of thermogenesis and adiposity.

Our study found that acute exposure to cold and thermoneutral temperatures significantly altered the proteome of iWAT. In female iWAT, 38 proteins were differentially regulated in response to the housing temperature change. These differentially regulated proteins were primarily associated with pathways related to mitochondrial dysfunction, ELF2 and ELF4 signaling, and mTOR. Previous studies have highlighted the dynamic nature of the adipose tissue proteome in response to various environmental and physiological stimuli (32–35). Our findings contribute to this expanding knowledge base by demonstrating the significant impact of acute temperature changes on the iWAT proteome. Previous studies investigating temperature-induced remodeling of adipose tissue have reported robust alterations in proteins related to mitochondrial function, lipid metabolism, and thermogenic activation, particularly under chronic cold exposure (36, 37). However, in contrast to studies showing pronounced upregulation of classical thermogenic markers like UCP1 and enzymes involved in fatty acid oxidation, our dataset revealed more modest changes, likely reflecting the limited duration of exposure and the unique physiological context of post-reproductive female mice.These differences underscore how the timing, duration, and physiological state (e.g., postpartum) can influence the extent and nature of adipose tissue remodeling. A detailed comparison of overlapping and distinct proteins between our findings and published datasets is provided in **Supplementary Table X**, highlighting both shared molecular responses and context-specific adaptations to thermal environment. These findings demonstrate that even brief, intermittent exposure to altered housing temperatures can induce lasting structural and proteomic changes in subcutaneous adipose tissue, particularly iWAT, without altering thermogenic capacity. This highlights the sensitivity of adipose tissue to early environmental cues and suggests that ambient temperature can serve as a significant modulatory factor in metabolic regulation. The observed proteomic shifts—impacting mitochondrial function, cellular stress responses, and nutrient-sensing pathways—may influence long-term susceptibility to obesity, insulin resistance, or altered energy balance, even in the absence of overt metabolic dysfunction.

Interestingly, a previous study using an untargeted metabolomics approach found dramatically distinct metabolic responses in BAT and WAT during acute cold exposure (38). This suggests that adipose tissues exhibit markedly different metabolic adaptations to cold stress. Furthermore, a recent study showed that modest changes in housing temperature led to 180 differentially expressed proteins in iWAT, many of which were involved in lipid metabolism and bioenergetics, including Ucp1, fatty acid synthase, and ATP-citrate lyase (18). This is particularly relevant, as it is widely acknowledged that there are sex differences in lipid metabolism, with females exhibiting greater rates of triglyceride synthesis in subcutaneous WAT compared to any WAT depot in males (39). In the current study, 38 proteins were found to be differentially expressed in female iWAT in response to housing temperature changes. However, we did not observe differences in the actual bioenergetics function of the adipose tissue between the cold and thermoneutral groups, which may be attributed to the shorter duration of temperature exposure and smaller sample size.

While this pilot study provides valuable insights into the lasting effects of short-term housing temperature manipulation on maternal adipose tissue, several limitations should be acknowledged. First, although proteomic profiling revealed significant alterations in iWAT, we did not perform downstream functional validation of these protein-level changes, such as Western blotting or pathway-specific assays, which limits mechanistic interpretation. Second, the duration of temperature exposure was relatively brief and limited to the periconceptional and gestational windows, which may have attenuated the magnitude of metabolic or transcriptomic effects compared to chronic exposure models. Third, all measurements were performed after the lactation period to avoid hormonal confounding, but this post-reproductive time point may have masked transient or earlier phenotypic differences. Additionally, sample size constraints limited our ability to detect more subtle differences in energy metabolism or mitochondrial function. The study also focused exclusively on female mice due to their longer exposure window, precluding assessment of potential sex-specific effects. Lastly, while offspring sex ratio was evaluated, postnatal growth and metabolic outcomes in the offspring are not included here and are being analyzed for a future report. These limitations underscore the need for expanded studies with larger cohorts, functional assays, and inclusion of offspring phenotyping to fully elucidate the long-term consequences of early thermal environmental exposures.

In summary, these findings highlight the complex and duration dependent responses of adipose tissue to temperature changes. Our findings contribute to the understanding of how acute temperature shifts impact adipose tissue dynamics and provide insights into the complex metabolic adaptations in response to environmental challenges. Further investigations with longer-term exposures and larger sample size could elucidate more nuanced metabolic responses and potential sex-specific differences in adipose tissue function under varying temperature conditions.

## Data Availability

The raw data supporting the conclusions of this article will be made available by the authors, without undue reservation.
